# Specifics of anticipatory competence of adolescents with intellectual disabilities

**DOI:** 10.1192/j.eurpsy.2025.1996

**Published:** 2025-08-26

**Authors:** A. Akhmetzyanova, T. Artemyeva, M. Ilyushina

**Affiliations:** 1 Department of Psychology and Pedagogy of Special Education, Kazan Federal University, Kazan, Russian Federation

## Abstract

**Introduction:**

Anticipation forms the basis of all human activity, enabling people to plan and carry out their activities, as well as to communicate and interact with others. Well-developed anticipatory abilities allow adolescents to successfully adapt to society, establishing effective communication with adults and peers.

**Objectives:**

To study the specifics of anticipatory competence in adolescents with intellectual disabilities.

**Methods:**

The study involved 40 adolescents (aged 12-15) attending educational institutions for children with disabilities and intellectual impairments (6A00.0, ICD-11). The research employed the following methods: the “Achenbach’s Questionnaire,” V.D. Mendelevich’s “Test of Anticipatory Competence,” V.P. Ulyanova’s “Anticipation of the Outcome of a Situation with Norm Violations,” and the authors’ method “Study of Anticipatory Competence of Adolescents” by A.I. Akhmetzyanova and T.V. Artemyeva.

**Results:**

The study results show that adolescents with intellectual disabilities are capable of predicting future situations. However, these children exhibit insufficient development in the key components of anticipatory competence: personal-situational, spatial, and temporal anticipation. Adolescents in this group experience difficulties in anticipating conflict situations in interpersonal relationships and in predicting others’ responses to their own behavior. The methods’ results indicate that adolescents with intellectual disabilities often exhibit spatial anticipatory incompetence: common traits in this group include general motor awkwardness and difficulties in spatial orientation (M=41.65; N=52); sometimes they cannot predict the timing of a stressful situation in advance and struggle with time orientation (M=39.45; N=42). While most adolescents with intellectual disabilities recognize and understand societal norms and rules, they face significant difficulties in accepting them (M=11.05; MAX=32).

**Conclusions:**

The data obtained in this study allow for the development of programs aimed at adapting adolescents to society, taking into account their ability to predict future situations and their anticipatory competence in various spheres: family relationships, virtual reality, and academic and extracurricular activities.

This paper has been supported by the Kazan Federal University Strategic Academic Leadership Program (PRIORITY-2030).

**Disclosure of Interest:**

None Declared

